# Standardizing the factors used in wind farm site suitability models: A review

**DOI:** 10.1016/j.heliyon.2023.e15903

**Published:** 2023-04-29

**Authors:** Joshua J. Wimhurst, Chinedu C. Nsude, J. Scott Greene

**Affiliations:** Department of Geography and Environmental Sustainability, University of Oklahoma, Norman, OK, 73019, USA

**Keywords:** Suitability analysis, Wind energy, Renewable energy siting factors, Thematic synthesis, Geographic information science, Multi-criteria decision analysis

## Abstract

As global wind energy capacity continues to expand, the need to site commercial wind farms in productive, affordable, and technically feasible locations has become increasingly important. The use of wind farm site suitability models to identify these locations has grown consequently, thus increasing interest in standardizing certain aspects of these models' development. This systematic review of wind farm site suitability studies seeks to identify similarities and differences in the selection and representation of their enlisted siting factors. The review focuses on how subjective modeling decisions, such as vocabulary choices and dataset selection, occur in the literature, based on five identified themes: 1) *Deciding Upon Siting Factors*, which explains how a study's geographical context, selected modeling approach, and modeler decisions can influence siting factor selection; 2) *Classifying Data and Siting Factor Terminology*, which addresses the extent and the advantages of consistent siting factor vocabulary; 3) *Implementing Siting Factors as Constraints or as Evaluation Criteria*, which covers the importance of consistent implementation and of specifying logic when enlisting siting factors to assess potential wind farm sites; 4) *Utilizing Primary and Secondary Data*, which details how a study's reliance on external or self-collected datasets influences siting factor representation; and 5) *Data Source and Accessibility*, which highlights the inconsistent provision of citations and dataset sources, and the availability of datasets for siting factors to the broader scientific community. Standardizing the selection and representation of siting factors would benefit comparisons between wind farm site suitability studies and communication of model outputs to a wider audience.

## Highlights

• Systematic review of siting factors in wind farm site suitability modeling studies.
• Selection and implementation of siting factors were summarized across five themes.
• Prior use, study context, and modeler preferences influence siting factor choice.
• Means of siting factor representation can influence suitable wind farm locations.
• Siting factor standards will be important as demand for wind energy research grows.

## Abbreviations

AHP: Analytic Hierarchy Process
ANP: Analytic Network Process
BWM: Best-Worst Method
ELECTRE, in French: ELimination Et Choice Translating REality
GIS: Geographic Information Systems
GW: Gigawatts
IBA: Important Bird Area
MCDA: Multi-Criteria Decision Analysis
TODIM, in Portuguese: Multicriteria Interactive Decision Making
VIKOR, in Bosnian: Multicriteria Optimization and Compromise Solution
OWA: Ordered Weighted Averaging
PROMETHEE: Preference Ranking Organization Method of Enrichment Evaluation
PRISMA: Preferred Reporting Items for Systematic Reviews and Meta-Analyses
TOPSIS: Technique for Order of Preference by Similarity to Ideal Solution
WiFSS: Wind Farm Site Suitability

## 1 Introduction

### 1.1 Overview

Global onshore and offshore wind energy capacity more than tripled during the 2010s, increasing from 220 Gigawatts (GW) in 2011 to 733 GW in 2020 [1]. This growth has made wind energy a significant contributor to many countries' electricity portfolios. In 2020, the European Union sourced 16.4% of its consumed electricity from wind turbines [2]. That same year, China's installed wind energy capacity accounted for 12.8% of its total 289 GW capacity [3]. The United States has experienced similar expansion, with approximately 9.2% of its electricity coming from the wind in 2021 [4]. This growth has occurred in accordance with wind energy's increasing appeal among governments [5,6], the private sector [7,8], and the general public [9,10].

Three drivers can explain most of the wind energy sector's recent appeal and expansion. Firstly, compared to fossil fuels, wind energy emits fewer air pollutants and greenhouse gases [[11], [12], [13], [14], [15]], and has lower water demands [16,17]. Hence, increasing wind energy production is often recommended to assist with climate change mitigation and sustainability strategies [[18], [19], [20]]. Secondly is wind energy's falling Levelized Cost of Electricity [[21], [22], [23]]; wind energy can increasingly compete with traditional energy sources [24,25] due to its declining capital, maintenance, and manufacturing costs [[26], [27], [28]]. Finally, many nations see wind energy's adoption as a means to achieve energy security [[29], [30], [31]], thereby diversifying energy generation options and reducing dependence on unstable or expensive foreign energy imports [[32], [33], [34]].

Many countries are taking deliberate steps to capitalize on these drivers, thus facilitating wind energy's continued deployment. Offshore wind energy's falling costs [[35], [36], [37]] have spurred interest in Western Europe, which has constructed the world's first floating offshore wind farms [38,39]. Similarly, the United States' recently enacted 30% investment tax credit [40], and 30 GW by 2030 capacity target [41] are projected to boost offshore wind energy development. Elsewhere, African countries such as Egypt, Morocco, and South Africa have legislated onshore wind energy capacity targets of their own [42], allowing them to leapfrog the fossil fuel-intensive development stage of more industrialized economies [43]. Wind energy's growing role in the electricity portfolios of countries around the world raises the question of where future commercial wind farms would be best installed.

### 1.2 Wind farm site selection

Selecting sites for wind farm installation is not as simple as building turbines in areas with high sustained wind speed. Physical barriers such as distance to grid transmission lines [44,45], ruggedness of terrain [46], presence of vulnerable flora and fauna [[47], [48], [49]], and proximity of infrastructure [[50], [51], [52]] can make commercial wind farms too expensive, challenging, or even dangerous to construct. There are also non-physical barriers to siting wind farms, ranging from investment and maintenance costs [53,54], to setback distances and other regulations [[55], [56], [57]], to public opinions about wind energy [[58], [59], [60]]. Improper wind farm siting decisions can have real-world consequences, such as high bird mortality rates in California's Altamont Pass [61] and Northern Portugal [62], as well as public opposition leading to project cancelations in Greece [63], Canada [64], and, famously, the United States' Cape Wind project [65,66]. As the wind energy sector grows in the coming decades, competition to construct commercial wind farms in profitable, low-impact locations will increase, thereby also increasing the salience of careful wind farm site selection.

This selection is frequently assisted by suitability analysis, “a process of systematically identifying or rating potential locations with respect to a particular use” [67]. An environmental model framework for suitability analysis yields a simplified representation of the factors that influence wind farm siting decisions, thus improving one's understanding of a system's behavior and outcomes [68,69]. Most wind farm siting models use a Geographic Information Systems (GIS) based approach [70], in which geospatial variables representing different siting factors are combined to form a composite suitability surface [71,72], with the variables typically weighted using Multi-Criteria Decision Analysis (MCDA), such as the Analytic Hierarchy Process (AHP) [[73], [74], [75]] or the Best-Worst Method [76,77]. GIS-based modeling approaches to Wind Farm Site Suitability (WiFSS) analysis have been performed in countries including Ecuador [78], India [79], Nigeria [80], Serbia [81], South Korea [82], Spain [83], the United States [84], to name a few. Alternative approaches to modeling WiFSS include Bayesian networks [85,86], logistic regression [[87], [88], [89]], and machine learning [90,91]. Although techniques for modeling WiFSS differ (e.g., Bayesian approaches quantify uncertainty in decision-making effectively but often lack the spatial explicitness of GIS approaches [92]), these techniques serve the common objectives of improving system understanding and informing the decision-making process for siting wind farms.

Despite these common objectives, WiFSS modeling approaches often vary in terms of their enlisted siting factors. Rediske et al. [93] summarized that certain siting factors are frequently enlisted in such studies, many of which describe physical features (e.g., wind speed, distance to roads/transmission lines/urban areas, land type, slope, etc.), though these factors are not all applied to every study. Siting factors may be excluded if their effects on siting decisions are perceived as lower [94], if they are covariant with other siting factors [95], or if they are simply irrelevant. Moreover, non-physical siting factors (such as project cost [96], government policies in effect [97], and demographics [98]) are harder to include in GIS-based approaches to WiFSS because of their need to be spatialized [99] for evaluation on a continuous domain. Non-physical siting factors are, however, often utilized in non-GIS-based approaches by analyzing expert opinions in order to rank candidate wind farm sites [[100], [101], [102]]. Siting factor selection is also contextual; some WiFSS studies, particularly GIS-based ones, are performed to assess wind farm locations based solely on features of the land itself [71,78,103]. Conversely, other studies situate their analysis within a broader social or economic context, such as wind farm project acceptability [89,104,105] or total project costs [[106], [107], [108]], thereby altering the enlisted siting factors. In short, although many factors can be considered relevant to wind farm siting decisions, some are incorporated more frequently into WiFSS models than others, and the same factors may be represented in different ways by different studies.

### 1.3 Objectives

Building models to inform wind farm site selection is a growing discipline, as evidenced by recent review papers [70,93,109]. This growth highlights the need for separate model developers to prioritize communication and knowledge sharing, to better ensure the continued refinement of WiFSS models and their potential benefits for policymaking [110]. Modeling collectives from other disciplines, such as the Coupled Model Intercomparison Project [111], encourage their participants to utilize a common set of variables and experiments in order to standardize climate model performance and facilitate comparisons of different models' outputs. Jakeman et al. [68] note that model building and usage are inherently subjective and benefit from standardization. Addressing this subjectivity, and thus recommending siting factor standards to be adopted in WiFSS modeling studies, is this review's key contribution to future suitability analyses.

This paper's objective is thus to examine the state of science related to the use of factors that can restrict or influence the siting of commercial wind farm projects in WiFSS models. Previous review articles have covered the wind farm site selection process [93], application of MCDA to siting renewable energy projects [109], and wind energy development's social [112,113] and environmental [16,114] impacts. This paper will focus specifically on contrasting how siting factors are selected and represented in WiFSS models, rather than describing their place in the broader modeling process, by performing a systematic literature review. This review endeavors to answer the following research question: how have previously conducted model-based studies of Wind Farm Site Suitability selected and represented their siting factors, and are there any overarching trends across the literature in this representation? A thematic synthesis of over 20 years of existing publications has been performed in order to identify these trends, discussed under the headings of five themes (Section 3). The paper will conclude by proposing an emphasis on standardizing these factors' selection, representation, and accessibility in future work (Section 4).

## 2 Method

### 2.1 Article search approach

This study was conducted by performing a systematic literature review with a thematic synthesis approach that grouped findings into themes [115], allowing for methodical article selection and thus reducing author bias in the review process [116]. This review sought articles about WiFSS models that detail the selection and representation of their siting factors, from which inconsistencies in how different studies selected and represented them could be deduced. Articles for this review were identified and extracted through snowballing and through database searches via Web of Science/Scopus, over the period from March 2022 to May 2022.

High-impact peer-reviewed journals published between January 2000 and May 2022 were sought for the database search, ensuring that this review consisted of contemporary articles up to the time of the search process. Existing reviews on related topics [70,93,109] took a similar approach when identifying potential articles. A modeling approach was not specified for the dataset search (e.g., MCDA, logistic regression, machine learning), because contrasting siting factor representation across modeling approaches was of interest to this review. Snowballing added to the database search by using the reference lists of existing publications, thus expanding the list of obtained articles for this review [117]. Specifically, a “backward snowballing” approach [118] for articles that mentioned WiFSS modeling in their titles and/or abstracts was enlisted. The reference lists of two review articles were used: Rediske et al.‘s [93] review of the wind farm site selection process, and Shao et al.‘s [109] review of MCDA applied to renewable energy site selection, both of which are relevant to the current topic and were published in high-impact journals.

### 2.2 Producing the final article list

**Fig. 1:**
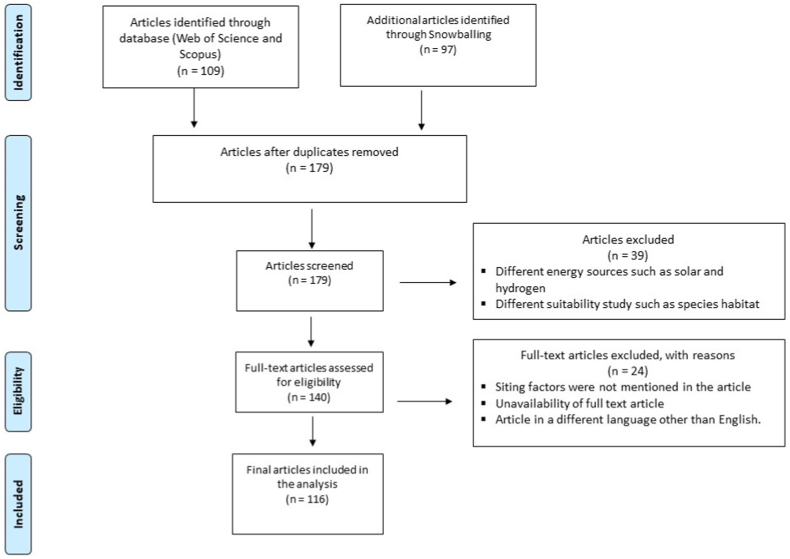
A PRISMA (Preferred Reporting Items for Systematic Reviews and Meta-Analyses) Flow Diagram that illustrates the method by which articles for this review paper were identified, screened, and finalized.

A PRISMA (Preferred Reporting Items for Systematic Reviews and Meta-Analyses) Flow Diagram [119] illustrating the refinement of the collected articles is shown in Fig. 1. The article search identified 206 articles in total, 109 of which came from the database search and 97 more from snowballing (54 articles from Rediske et al. [93] and 43 from Shao et al. [109]), 27 of which were duplicates identified by both approaches. The title, abstract, and keywords of the 179 non-duplicate articles were screened for references to wind energy or site suitability analysis. This screening removed 39 articles that focused on other sources of energy (e.g., solar, tidal, geothermal) or on assessing the suitability of non-energy systems. A full-text assessment of the remaining 140 articles sought details about siting factors for a suitability analysis and/or techniques associated with WiFSS modeling (e.g., MCDA). This full-text assessment removed 20 articles that did not specify their siting factors, three articles lacking a full-text version (despite requests from their authors), and one article not written in English. The PRISMA approach left 116 articles eligible for inclusion in the current review.

### 2.3 Data extraction and overviewing the included articles

**Fig. 2:**
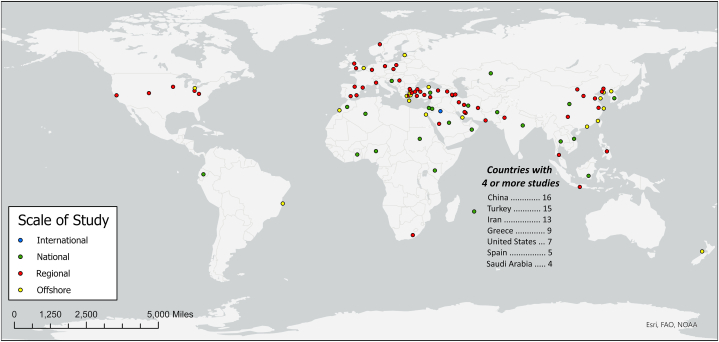
Study locations of the articles included in the systematic literature review. Points are colored by the spatial scale of the study performed. Note that some points overlap due to studies being performed over the same spatial domain. Basemap from Esri [121].
3) *Onshore or Offshore,* for documenting whether articles assessed onshore or offshore WiFSS. Offshore WiFSS studies accounted for 21 (18%) of the 116 articles included in this review (offshore study locations in Fig. 2 (yellow points) are based on their approximate centroids). The number of offshore WiFSS studies is sufficiently large to allow for the assessment of onshore versus offshore siting factor differences.
4) *Modeling Approach*, for summarizing the type of model and factor ranking/weighting methods enlisted by each article. Table 1a shows that 98 (85%) of the 116 reviewed studies enlisted an MCDA approach for assessing WiFSS, with AHP being the most common (51 out of 98 studies), and most studies being performed in a GIS-based environment (81 out of 98 studies) that utilized secondary datasets (e.g., digital elevation models, land cover rasters, census statistics, etc.). Conversely, 18 (16%) studies used a non-MCDA approach (Table 1b), with Data Envelopment Analysis (DEA) and GIS-based models being frequent choices. The types of data collected for WiFSS models are often connected to the modeling approach, e.g., non-GIS-based MCDA approaches that collect and weight expert opinions about siting factors to rank candidate wind farm sites [100], [101], [102], [187], [188], [189], [190], [191], [192], [193], [194]. As such, modeling approach can affect the selection and representation of siting factors.
5) *Basis for Factor Selection*, for documenting how each reviewed article decided upon its siting factors, with all studies relying on at least one of four methods. Eighty (69%) of the 116 studies used previous literature to justify their siting factor choices, 38 (33%) studies relied on their authors' opinions about which factors to include, 24 studies (21%) enlisted external expert opinions to inform these decisions, and 20 studies (17%) stated that siting factor selection was influenced by knowledge of local geography and/or legislation.

A spreadsheet was created using the articles identified by this systematic review (see Supplementary Material), which compiled each article's siting factors, along with other relevant information under the column headings described below:

1) *Year of Publication*, for ensuring that the methods of siting factor selection and representation summarized in this review are contemporary. Of the 116 included articles, 66 of them (41 from the database search, 25 from snowballing) were published from 2018 to 2022.
2) *Country of Origin*, for illustrating the case study contexts of these articles. These contexts are illustrated in Fig. 2, showing a large number of studies from China (16), Turkey (15), Iran (13), Greece (9), the United States (7), Spain (5), and Saudi Arabia (4). Studies were also conducted on multiple spatial scales, whether regional (city/county/state), national, or international; country and spatial scale could impact the treatment of siting factors.

Because this review's interest is in examining siting factor differences across WiFSS studies, several properties of all siting factors enlisted in each reviewed article were documented in this review's Supplementary Material. These properties consisted of “File Type” (Vector, Raster, Point Observations, Unspecified), “Factor Type” (Constraint, Evaluation, Unspecified), “Constraint Nature; Logic” (if the factor was implemented as a constraint, how was it implemented, and by what logic), “Data Source” (the primary or secondary data that supplied the siting factor, e.g., expert opinions, a website source, legislation), “Classification” (Economic, Environmental, Social, Technical, etc., otherwise Not Classified), and “Combined Factors” (describes grouped sub-factors, and/or siting factor names that represent the same concept across different studies). Each of these six properties underpins the thematic synthesis presented in the current review [115], and the Sections in which each theme is discussed are accompanied by figures and tables that provide bibliometrics for each of these properties [120]. For instance, the “Classification” property is critical to Section 3.2, with bibliometrics computed that quantify the commonest language (e.g., technical, economic, environmental, etc.) used to classify siting factors across all reviewed articles. Absent information about siting factor properties was documented in the Supplementary Material as “N/A”, for example, an unspecified data source or file type for a particular siting factor. .

**Table 1a:** Articles included in the systematic literature review, for MCDA approaches only. Acronyms for the listed MCDA methods: Analytic Hierarchy Process (AHP), Analytic Network Process (ANP), Technique for Order of Preference by Similarity to Ideal Solution (TOPSIS), Multicriteria Optimization and Compromise Solution (VIKOR, in Bosnian), Ordered Weighted Averaging (OWA), Best-Worst Method (BWM), ELimination Et Choice Translating REality (ELECTRE, in French), Preference Ranking Organization Method of Enrichment Evaluation (PROMETHEE), Multicriteria Interactive Decision Making (TODIM, in Portuguese).

MCDA Approach	Study Context	Data Type (Secondary/Primary)	Weighting/Ranking Method	References
GIS-based	Onshore	Secondary	AHP	[[46], [74], [75], [78], [79], [80], [82], [83], [94], [104], [122], [123], [124], [125], [126], [127], [128], [129], [130], [131], [132], [133], [134], [135], [136], [137], [138], [139], [140], [141], [142], [143], [144], [145], [146], [147], [148]]
			ANP	[[149], [150], [151]]
			TOPSIS	[77,78,83,135,145]
			VIKOR	[77,78]
			OWA	[78,122,132,133,[151], [152], [153]]
			BWM	[76,77,154]
			Prescribed Weights	[84,[155], [156], [157], [158], [159]]
			No Scheme/Equal Weights	[[160], [161], [162], [163], [164], [165], [166], [167], [168]]
			Other	[45,[169], [170], [171]]
		Primary and Secondary	AHP-VIKOR	[72,172]
			TOPSIS	[173]
	Offshore	Secondary	AHP	[[174], [175], [176], [177], [178], [179], [180], [181], [182]]
			TOPSIS	[177]
			Prescribed	[183,184]
			No Scheme/Equal Weights	[185]
Non-GIS-based	Onshore	Secondary	AHP	[186]
		Primary	AHP	[100,187,188]
			VIKOR	[100]
			PROMETHEE	[102]
			ELECTRE	[189,190]
			TOPSIS	[101,191]
			Intuitionistic Fuzzy Logic	[192]
		Primary and Secondary	No Scheme/Equal Weights	[193]
	Offshore	Secondary	AHP	[194]
			TODIM	[195]
			No Scheme/Equal Weights	[196]
		Primary	BWM	[197]
			PROMETHEE	[198]
			ANP	[198,199]

## 3 Results fro m the thematic synthesis

Similarities and differences in the selection and representation of siting factors in WiFSS studies, based on this systematic review, can be summarized across five themes. These themes were developed based on the outcomes of the data extraction process described in Section 2.3, and the topics relating to siting factors mentioned in previous review papers [70,93,109]. The first theme, “Deciding upon Siting Factors” (Section 3.1), focuses on how study context, modeling approach, and author preferences result in some siting factors being enlisted more frequently than others. “Classifying Data and Siting Factor Terminology” (Section 3.2), the second theme, addresses the importance of consistent terminology and classification schemes (e.g., economic, environmental, social, technical) for siting factors across WiFSS studies. Some GIS-based WiFSS studies implemented siting factors as constraints on wind energy development, others for evaluation of development potential, which is covered by the third theme of “Implementing Siting Factors as Constraints or as Evaluation Criteria” (Section 3.3). The fourth theme is “Utilizing Primary and Secondary Data” (Section 3.4), which covers differences in siting factor representation using self-collected or external datasets, and how these dataset decisions vary with modeling approach. The importance of documenting data sources for siting factors, and the extent of doing so across WiFSS studies, comprises the final theme, “Data Source and Accessibility” (Section 3.5). A thematic synthesis evades discussion of each individual reviewed article, allowing Section 3 to focus instead on broader trends and features of all articles listed in Table 1a and 1b.

**Table 1b:** Same as Table 1a but for Non-MCDA approaches only.

Model Approach	Study Context	Data Type (Secondary/Primary)	References
Artificial Neural Network	Onshore	Secondary	[200]
Benefit-Cost Analysis	Offshore	Secondary	[201]
Data Envelopment Analysis	Onshore	Secondary	[[202], [203], [204], [205]]
		Primary and Secondary	[206,207]
GIS - Boolean Logic	Onshore	Secondary	[208]
GIS - Correlation Analysis	Onshore	Secondary	[209]
GIS - Least Cost Distance	Onshore	Secondary	[210]
Ideal Matter-Element Model	Onshore	Primary	[211]
Logistic Regression	Onshore	Secondary	[212]
Machine Learning	Onshore	Secondary	[90]
Maximum Entropy Model	Onshore	Secondary	[213]
Mixed Integer Linear Programming	Onshore	Secondary	[214]
Picture Fuzzy Modeling and TOPSIS	Offshore	Primary	[215]
Wind Atlas Analysis and Application Program	Onshore	Secondary	[216]

### 3.1 Deciding upon siting factors

**Fig. 3:**
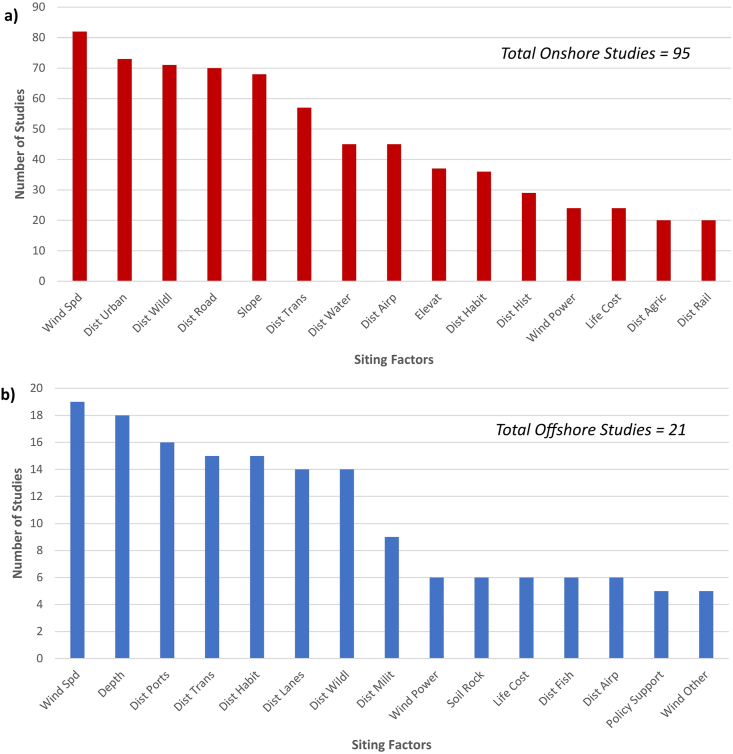
The 15 most commonly enlisted siting factors in the onshore (3a, top) and offshore (3b, bottom) WiFSS (Wind Farm Site Suitability) studies included in this systematic review. Refer to Table 2 for full factor names.

Siting factor decisions are often motivated by siting factors used in prior WiFSS studies. Doing so ensures that current modeling studies do not exclude important siting factors, while also facilitating model output comparisons for the same spatial contexts. Fig. 3 shows that, among the 116 reviewed articles (95 onshore WiFSS studies in Fig. 3a and 21 offshore WiFSS studies in Fig. 3b), siting factors that describe wind resources (e.g., Wind Speed, Wind Power Density; note that capitalized siting factors designate those identified by the systematic review), natural limitations (e.g., Slope, Elevation, Ocean Depth), and distance to land features (e.g., Distance to Roads/Transmission Lines/Protected Areas/etc.) are often selected, suggesting that WiFSS studies value consistent siting factor choices. This consistency continues in how WiFSS studies detail their selected siting factors. Eighty-five (73%) of the 116 reviewed articles summarized siting factors in table form (see Supplementary Material), with table columns typically detailing each factor's description [76,201], dataset source [163,181], citations [81,135], and implementation for constraint or evaluation [74,78]. Older WiFSS studies, such as Baban and Parry [161] and Rodman and Meentemeyer [71], along with recent, high-impact studies [104,155], are often cited to justify siting factor choices, establishing a frequently emulated style of factor selection and tabular presentation [143,148,150,213].

Fig. 3 also demonstrates how some siting factors are enlisted more consistently for WiFSS studies than others. Apart from Life Cycle Cost (24 onshore studies, six offshore studies) and Policy Support (five offshore studies), non-physical siting factors are absent among those most frequently enlisted. Malczewski [99] refers to non-physical siting factors as being “implicitly spatial,” meaning they have potential to be expressed in a spatial context, which non-GIS-based MCDA approaches realize by having experts rate the importance of siting factors, and then re-expressing these ratings numerically [173]. This rating and ranking of candidate wind farm sites lacks common siting factors across studies. Presumably important factors such as Elevation [188,191] and Distance to Airports [190,191,196] are enlisted by few of the non-GIS-based MCDA studies in Table 1a, and some of these studies excluded Wind Speed [101,187,189,193]. Such studies sometimes establish a context of ranking wind farm sites based on social acceptability [193] or economic viability [188,195], hence implicitly spatial data for electricity demand [100,190,191,193,195,198], local attitudes to wind farms [100], [101], [102], [188], [189], [195], [197], [198], [199], noise pollution [100,101,193,197,199], and other non-physical factors are more often selected. Some other non-GIS-based MCDA studies propose a general model framework without a case study [186,196,198], allowing selected siting factors to remain generalized for other modelers to implement. Ultimately, the siting factors used in non-GIS-based MCDA approaches to WiFSS depend on the study context, hence their less frequent application among the studies in this review.

The context dependence of siting factor choices also applies to GIS-based WiFSS studies. This context may simply be a study's objective, such as Gkeka-Serpetsidaki and Tsoutsos [181] examining social acceptance of offshore wind farm sites in Crete, using distance to the shoreline as a proxy for noise pollution and visual disturbance. Similarly, Vinhoza and Shaeffer [180] assessed offshore wind's economic attractiveness in Brazil by using distance to shipping ports to represent development costs. Siting factor inclusion may also be justified by geographical context, such as Díaz-Cuevas [131] including proximity to tourist facilities due to South Spain's large tourism sector, or Ouammi et al. [162] not considering distance to water bodies because those in Italy's Savona Province do not obstruct eligible wind farm sites. Pamučar et al. [76] and Sánchez-Lozano et al. [169] both noted that siting factor choices depend on the geographical area in question. Study context also explains why wind speed was not included in some of the reviewed articles (101 out of 116, Fig. 3). Wind speed may be deemed invariable across a small study domain [161,171], a study's authors may instead use wind power density to represent the wind resource [101,152,153,176,189,205,212], or including wind speed may not assist the study's objective [109,193]. Siting factor decisions therefore also depend on decisions made by a study's authors [82,94], not just geographical context and the chosen modeling approach.

Fig. 3b shows that, of the 21 offshore studies in this review, many enlisted Wind Speed (19) Ocean Depth (18), Distance to Shipping Ports (16), Distance to Transmission Lines (15), and Distance to Animal Habitats (15) as siting factors, suggesting some level of consensus about important siting factors for offshore WiFSS studies. However, siting factors that have documented relevance to offshore wind energy, such as Natural Disaster Risk [[217], [218], [219]] and Distance to Commercial Fishing Areas [[220], [221], [222]], were not as frequently used in these 21 studies, featuring in four [190,197,198,201] and six [176,178,180,183,185,194] studies, respectively. The exclusion of these factors could be due to a lack of relevant datasets or the relatively small amount of offshore WiFSS literature; the oldest offshore WiFSS study in this review was published in 2013 [201], compared to 2001 for onshore WiFSS studies [160]. The importance of prior WiFSS studies for deciding upon siting factors will likely increase, as will the incorporation of underused siting factors as the demand for offshore wind energy research grows.

### 3.2 Classifying Data and Siting Factor Terminology

WiFSS modeling studies often classify their siting factors by grouping them under terms such as environmental [45,78,156,164,165,201], economic [122,143,145,166,174,213], social [81,94,124,129,180,211], and technical [79,100,123,140,175,202]. Classification allows for vocabulary control when describing siting factors with similar effects on WiFSS. For example, the distance to the nearest city, transmission line, or road all present a common technical limitation to wind farm siting [131,140]. Additionally, environmental limitations are posed by the noise pollution and visual impact associated with wind turbines [82,152]; hence, these siting factors are similarly classified together. A second benefit of classification in WiFSS studies is organizing one's analysis. Siting factors classified as environmental or technical often serve as constraints, such as limited development in protected wildlife areas [104,155,164], land that is too elevated or steep [79,140,142], or areas with insufficient wind speeds [123,139,178]. Similarly, economic siting factors like land leasing and maintenance costs are often incorporated into WiFSS models within a subset of equations that calculate cost competitiveness of candidate wind farm sites [122,165,177,200,214], hence their common classification.

Table 2a presents the classification terms most utilized across this systematic review. For both onshore (Table 2a) and offshore (Table 2b) studies, Distance to Animal Habitats or Migration Routes was most frequently classified as an environmental factor [163,180,181,198], as were other factors that relate to natural land features, such as Distance to Protected or Wildlife Areas [131,140,149,160] and Distance to Water Bodies [90,150,160,164]. This consistency was lacking for other siting factors, with different studies classifying Wind Speed as a technical [131,140], economic [94,168], environmental [81,142], or climate [46,77] factor. Similar inconsistency exists for siting factors relating to distance from infrastructure. Table 2 shows studies commonly classifying Distance to Airports [150,156] Distance to Urban Centers [81,164] and Distance to Railroads [131,164] as environmental factors, whereas Distance to Roads [82,152], Distance to Agricultural Areas [142], and Distance to Transmission Lines and Substations [142,152] were often classified as economic factors. Inconsistent classification is further complicated by the prescribed influence of siting factors. For example, some studies [45,142] prescribe proximity to population centers as a social and economic asset (lower construction costs; closer to demand areas), but other studies [82,152] prescribe this proximity as a social and economic detriment due to increased noise pollution and visual impact from new wind farms. The classification terms adopted for the siting factors of WiFSS models therefore depend to an extent on the subjective decisions of model developers.

**Table 2a:** Language used to classify the 15 most common siting factors in the onshore WiFSS studies included in this systematic review. The number of studies including each factor that did not use classification is also given. See the “Classification” columns of the Supplementary Material.

Siting Factors	Common Classifications (Frequency)	Studies Without Classification
Wind Speed	Technical (15); Economic (14); Wind/Weather (12); Climate (7); Environmental (5)	30
Distance to Protected or Wildlife Areas	Environmental (37); Social (3); Protective (2); Location (2)	28
Slope	Economic (10); Technical (8); Topography (7); Geographical (5); Environmental (3)	28
Distance to Urban Centers	Environmental (14); Social (14); Planning (6); Economic (5); Technical (3)	26
Distance to Roads	Economic (26); Environmental (7); Technical (7); Location (3); Planning (3)	24
Distance to Transmission Lines or Substations	Economic (17); Technical (5); Environmental (3); Infrastructural (3); Location (2)	24
Distance to Airports	Environmental (9); Economic (5); Protective (3); Location (2); Political (2)	20
Distance to Water Bodies	Environmental (23); Social (3); Economic (2); Location (2); Protective (2)	15
Elevation	Technical (6); Environmental (5); Topography (5); Economic (3); Geographic (2)	15
Distance to Animal Habitats or Migration Zones	Environmental (21); Social (3); Protective (2)	15
Distance to Agricultural Areas	Economic (2); Technical (2)	14
Distance to Historic Places	Environmental (10); Social (4); Cultural (2)	11
Distance to Railroads	Environmental (4); Economic (2)	11
Wind Power Density	Wind/Weather (8); Technical (4); Climate (2)	10
Life Cycle Costs	Economic (13); Technical (3); Social (2)	8

Another example of this subjectivity is the decision not to use a classification scheme. Table 2b shows that, of the 21 offshore WiFSS studies, six (29%) studies that included Distance to Shipping Lanes did not classify their siting factors [176,178,183,184,190,215]. Of these six studies, Wu et al. [190] and Zhang et al. [215] are non-GIS-based approaches (of the 32 non-GIS-based studies in this review, eight studies did not classify their siting factors [186,[204], [205], [206], [207],^212^,^214^,^216^], and eight studies classified only some of them [100,190,194,196,197,199,203,215]). The other four studies that included Distance to Shipping Lanes are GIS-based, despite Tercan et al. [178] stressing the importance of having technical, economic, environmental, and social criteria for evaluating potential offshore wind farm sites. An absent classification scheme sometimes appeared concurrently with other modeling decisions. Of the 32 studies in this review that did not classify Wind Speed (Tables 2a and 2b), GIS-based studies that included an equation-based economic/technical analysis of potential wind farm locations often lacked a classification scheme [76,82,146,151,162,165,168], as did studies with a small number (less than seven) of siting factors [125,126,159,186,200,204,208]. The application of classification schemes across WiFSS studies is inconsistent and not well-defined, thus making the intended role of siting factors when comparing WiFSS study approaches potentially unclear.

**Table 2b:** Same as Table 2a but for the offshore WiFSS studies included in this systematic review.

Siting Factor	Common Classifications (Frequency)	Studies Without Classification
Distance to Shipping Ports or Coastlines	Economic (5); Local Conditions (3); Technical (2); Region Characteristics (1); Social (1)	6
Distance to Shipping Lanes	Political (2); Protective (2); Social (2); Technical (2); Environmental (1)	6
Distance to Animal Habitats or Migration Routes	Environmental (10); Protective (1)	5
Distance to Protected or Wildlife Areas	Environmental (8); Protective (2)	5
Ocean Bathymetry	Economic (5); Technical (5); Construction (2); Sea State (2); Region Characteristics (2)	4
Distance to Transmission Lines or Substations	Local Conditions (4); Economic (3); Technical (2); Region Characteristics (1); Safety (1)	4
Distance to Military Zones	Protective (2); Environmental (1); Political (1); Region Characteristics (1); Safety (1)	4
Distance to Commercial Fishing Areas	Social (2)	4
Wind Speed	Technical (9); Wind/Weather (6); Economic (4); Geographical (1)	2
Wind Power Density	Wind/Weather (4)	2
Life Cycle Costs	Economic (4)	2
Distance to Airports	Local Conditions (1); Environmental (1); Safety (1); Technical (1)	2
Soil or Rock Type	Construction (1); Environmental (1); Region Characteristics (1); Sea State (1); Technical (1)	1
Other Wind Properties (Turbulence, Effective Wind Hours, Direction)	Wind/Weather (3); Environmental (1)	1
Policy Support	Social (3); Cultural (1); Economic (1); Safety (1)	0

This lack of clarity also comes from WiFSS studies using different terminology to describe the same siting factors. Wind Speed was referred to by several different terms throughout the systematic review, such as “Wind Potential” [84,104], “Wind Sources” [195], “Average Wind Blow” [202], and “Efficiency” [100]. It can be implied that these terms describe the same siting factor, but not for certain, and this uncertainty increases if the means of data collection for each WiFSS study is different, e.g., a downloadable wind speed dataset versus expert opinion about wind speed's importance. Conversely, some studies used the same terminology to describe different siting factors; the term “Protected Areas” was used to describe forests [90,130,138], bird habitats [81,137,201], marine habitats [175,184,190], or combinations of these features. Common language for both describing and classifying siting factors is essential for any modeling discipline, such as climate modeling [111], especially given the number of recently published WiFSS studies (see Supplementary Material). WiFSS modeling would benefit from nomenclature for siting factor terms and their classification, thereby assisting communication when using prior literature to inform studies, and establishing a standard language for model developers to adopt [110].

### 3.3 Implementing Siting Factors as constraints or as evaluation criteria

The implicitly geospatial nature of non-physical siting factors [99] means that their continuous variation in space requires being placed onto a gridded dataset (see Mann et al.‘s [212] approach to census demographics, or Brewer et al.‘s [223] to social attitude surveys), or being proxied with a physical siting factor, such as inferring noise pollution or visual impact based on distance from infrastructure [130,145,152,157,181,210]. Either of these approaches allow non-physical siting factors to be treated alongside the datasets that commonly represent physical siting factors in GIS-based WiFSS models, such as line shapefiles of powerlines for transmission line proximity [74,80,136,144,164,179] or rasters of wind speed for assessing the resource itself [78,79,90,126,130,201]. The common function of the datasets for each siting factor is to inform a GIS-based model's assessment of wind farm potential across a continuous spatial domain. Where GIS-based WiFSS studies frequently differ is in their implementation of the same siting factors as constraints and/or evaluation criteria.

In the context of the papers studied here, constraints are Boolean restrictions that eliminate potential wind farm locations based on a minimum standard [80,155,164], such as land being too steep [74,156,161], being too close to historic landmarks [135,145,181], among many others. These constraints are typically either a buffer distance around land features (e.g., no wind farms within 500 m of a river [142]), or prohibition within an area of conflicting land use (e.g., no wind farms in a designated vulnerable bird habitat [83]). Evaluation criteria assess WiFSS outside the constrained zones, either as an ordinal [102,134,159] or a quantitative [179,184,208] value. The two commonest types of evaluation criteria are those that assess suitability with distance from physical features (e.g., WiFSS being greater closer to roads [75]), and those based on magnitude at a singular point in space (e.g., WiFSS being greater in high-altitude locations up to 2000 m [46]). Table 3a summarizes onshore (Table 3a) and offshore (Table 3b) physical siting factors that were frequently enlisted as constraints and/or evaluation criteria by the studies in this review, with factors representing distance to land features (e.g., Distance to Urban Centers, Distance to Shipping Ports or Coastlines, Distance to Protected or Wildlife Areas), Slope, Wind Speed, and Ocean Depth being especially common as constraints. Some of these studies used siting factors to both constrain and evaluate WiFSS, such as Ajanaku et al. [140] excluding areas of West Virginia more than 10 km from transmission lines (constraint), and having suitability increase with proximity to transmission lines outside of constrained zones (evaluation). Many siting factors were used to both constrain and evaluate WiFSS across the studies in this review, particularly Wind Speed [ 125,143,144], Distance to Roads [80,136,139], and Ocean Depth [178,180,201], hence the high counts in both columns of Table 3 for these siting factors.

**Table 3a:** Number of studies that employed siting factors as constraints and/or evaluation criteria, among the most common siting factors used in the onshore WiFSS studies included in this systematic review. The frequency of unspecified logic for constraint criteria for each siting factor is also given in parentheses. See the “Factor Type” and “Constraint Nature; Logic” columns of the Supplementary Material.

Siting Factor	Frequency as Constraints (Unspecified Logic)	Frequency as Evaluation Criteria
Distance to Urban Centers	57 (15)	38
Distance to Protected or Wildlife Areas	52 (10)	23
Slope	51 (14)	42
Distance to Roads	49 (24)	51
Wind Speed	47 (18)	70
Distance to Airports	38 (14)	13
Distance to Water Bodies	37 (9)	12
Distance to Transmission Lines or Substations	30 (13)	42
Distance to Animal Habitats or Migration Zones	29 (9)	13
Distance to Historic Places	24 (7)	7
Elevation	21 (8)	20
Distance to Railroads	16 (4)	4
Distance to Agricultural Areas	9 (4)	14
Wind Power Density	7 (4)	18

**Table 3b:** Same as Table 3a but for the offshore WiFSS studies included in this systematic review.

Siting Factor	Frequency as Constraints (Unspecified Logic)	Frequency as Evaluation Criteria
Ocean Depth	14 (1)	13
Distance to Shipping Ports or Coastlines	12 (1)	10
Wind Speed	11 (0)	13
Distance to Animal Habitats or Migration Routes	11 (0)	2
Distance to Protected or Wildlife Areas	11 (1)	2
Distance to Shipping Lanes	9 (0)	5
Distance to Military Zones	9 (0)	1
Distance to Transmission Lines or Substations	9 (2)	7
Distance to Airports	5 (0)	2
Distance to Commercial Fishing Areas	5 (0)	1
Soil or Rock Type	4 (1)	3
Wind Power Density	2 (2)	5
Other Wind Properties (Turbulence, Effective Wind Hours, Direction)	1 (1)	4

Despite these similarities in the implementation of constraints and evaluation criteria across WiFSS studies, this review highlighted some important differences:

1. *Specifying logic for the selection and setting of constraints.* Most studies in this review utilized existing legislation (e.g., laws prohibiting wind energy development in protected areas [176]), previous WiFSS modeling studies (e.g., setting a maximum land slope based on a prior study [154]), and/or a chain of reasoning in the main text (e.g., setting a buffer around airports to mitigate radar signal interference [170]) to justify the selection and setting of constraints. Some studies, however, did not provide logic for their models' constraints. Table 3a shows that, of the 49 studies that implemented Distance to Roads as a constraint, 24 (49%) did not justify this constraint in any of the manners mentioned above. Unspecified logic was, conversely, significantly less common for offshore siting factors (Table 3b). Not justifying constraints leaves readers to guess whether a constraint is appropriate, and furthermore whether the constraint is transferable to other contexts. For instance, a 300-m buffer distance around railroads might be acceptable for a WiFSS study in Northwest Iran [ 164], but whether 300 m would be acceptable for studies in other locations is uncertain due to absent logic.
2. *Inconsistent implementation of constraints.*Table 3 suggests a common set of constraints employed by WiFSS studies, such as a minimum wind speed [123,167,185,195,214], limiting wind farm construction in protected areas [71,74,78,143,154], a minimum distance from urban centers [149,152,161,181,213], to name a few. However, the magnitude of these common constraints varies widely. Pamučar et al. [76] enlisted a maximum land slope of 7%, in contrast to Tegou et al.‘s [123] constraint of 30% (some studies instead constrained land slope with a degree angle [46,94,164], adding further inconsistency). Additionally, for distance-based constraints, some studies enlisted a prohibition rather than a buffer. Whereas Cradden et al. [184] only prohibited wind energy development in Europe's Important Bird Areas (IBAs), Ayodele et al. [80] also included a 300-m buffer around Nigeria's IBAs, despite both studies using the same dataset [224]. A third facet of inconsistent implementation is the exclusion of important constraints. Compared to factors listed in Table 3, few studies in this review enlisted constraints for Distance to Mines or Pits [131,144,155], or Distance to Fault Lines [150,165,168], despite the known risks of building wind farms in earthquake-prone areas [225,226] and over mines [227]. The decision to implement specific constraints in WiFSS models is sometimes context-dependent (e.g., there is no need to include fault line proximity if the study area does not experience earthquakes), but constraints having a consistent magnitude, units, and nature (prohibition or buffer distance) across studies is nevertheless important.
3. *Enlisting siting factors as constraints or evaluation criteria in different studies.* While some studies enlist siting factors to both constrain and evaluate WiFSS, as previously discussed, other studies may enlist a siting factor only for constraint or only for evaluation. For instance, Mekonnen and Gorsevski [183] and Kazak et al. [157] set WiFSS to increase with distance away from bird habitats, with no specified minimum distance or similar constraint. Conversely, Değirmenci et al. [130], Genç et al. [185] and Ouammi et al. [162] implemented proximity to bird habitats and migration routes strictly as a constraint. An increasing suitability with distance from a bird habitat (i.e., evaluation) would produce a different model output than just buffering the same habitat (i.e., a constraint), resulting in two different WiFSS outputs for the same spatial context. This difference presents a planning risk, knowing the negative impacts of improper wind farm siting on avian species [61,62]. The decision to implement siting factors for either constraint or evaluation may be motivated by usage of these factors in prior studies, and/or the modelers' objectives, for example, the evaluation of wind resources in remaining locations after applying all other siting factors as constraints [162,[164], [165], [166], [167],^214^].

The implementation of siting factors as constraints or evaluation criteria can be subjective, again depending on a WiFSS study's context and individual modeler preference. Addressing this subjectivity could benefit the consistency of GIS-based WiFSS studies by normalizing the use of literature and legislation to inform the magnitude of constraints, thus explicitly justifying the use of specific siting factors for constraint and/or evaluation. This implementation can also depend on regulations observed in a particular country or region. For example, some counties in the United States enforce setback distance constraints on wind energy development, other counties do not, and in some cases these constraints may instead be enforced at higher levels of government [56].

### 3.4 Utilizing Primary and Secondary Data

Table 1a and 1b shows that most studies in this review utilized secondary datasets, particularly those with GIS-based MCDA approaches, usually in the form of downloaded geospatial data [81,90,124,145,181] and previously recorded observations [80,125,147,162,206]. Some secondary datasets were enlisted by multiple studies, such as road information obtained from OpenStreetMap [130,136,144], Digital Elevation Models from the United States Geological Survey [71,80,133], and wind speed information from the Global Wind Atlas [79,143,148]. The use of such datasets for WiFSS studies exemplifies the value of free resource access for public sector model development [228], because developers are thereby encouraged to use a common set of siting factors, facilitating standard language and comparisons of modeling approaches that are less biased by siting factor choices. Differences in model outputs for the same geographical contexts could indeed be partially attributed to their enlisted secondary datasets. For instance, WiFSS studies from Turkey that represented protected areas with secondary datasets enlisted data either from the state government [134,138] or from larger organizations such as the European Environment Agency [213] and the United Nations Educational, Scientific and Cultural Organization [130]. Each data source has its own unique definition of protected areas, which combined with modeler preferences results in quite different depictions of protected areas for the same country. Selected secondary datasets therefore have important consequences for the consistency and comparability of model outputs across (GIS-based) WiFSS studies.

By contrast, primary data were enlisted almost entirely by non-GIS-based MCDA studies in this review (Table 1a). These primary data are usually the collected opinions of academic or industrial experts, whether from questionnaire responses [102,187,188,192,197,199], conducted interviews [101,193], or focus groups [191,195,198], with the objective of assessing discrete wind farm sites based on the rated importance of a set of siting factors. These ratings are usually ordinal and employ either a linguistic scale to express each siting factor's individual importance [100,101,187,190,191,193,198,199], or a ranking of siting factors relative to each other [102,188,197]. There are multiple examples in this review of both physical and non-physical siting factors being enlisted by WiFSS studies that relied solely on primary data (e.g., studies that enlisted both Wind Speed/Power Density and Life Cycle Cost [101,102,192,197,199]), because they are both collected using opinion-based methods. However, the siting factors incorporated into these studies are not consistent. Gamboa and Munda [193] and Aras et al. [187] excluded Wind Speed due to their interest in the social and technical feasibility of wind farm sites, respectively, and very few of the studies examined here enlisted important siting factors like Distance to Military Zones [190,197] and Distance to Water Bodies [191]. Differences in siting factor choices could result in inconsistent wind farm site characterization, which can be a problem when comparing non-GIS-based MCDA studies with the same geographical contexts, such as those from China [[188], [189], [190],^192^,^194^,^195^,^198^] or Turkey [100,187,191,197]. The collection and application of primary data in these studies varied in other important ways, such as some studies using outside expert opinions, rather than those of the authors alone, to help decide upon siting factors [188,190,192,194,195,197,198]. Additionally, most studies applied fuzzy logic to quantify expert opinions and also to address the uncertainty inherent to linguistic decision-making [191], though some studies did not [102,[187], [188], [189],^197^]. Much like with secondary data, there are several conventions in the use of primary data to represent siting factors in WiFSS models, such as the use of linguistic scales and enlisting expert opinions, though these conventions are not universal.

A small number of studies in this review presented models that combined primary and secondary data in their assessment of WiFSS [72,172,173,193,206,207], eliminating the need for proxies or dataset transformations. For instance, Rezaei-Shouroki et al. [206] combined secondary wind speed observations and primary opinions about land price into the same set of Data Envelopment Analysis equations. Studies that enlist primary and secondary data also often take different approaches to WiFSS modeling, such as Xu et al. [72], which used datasets of bird migration routes and power plant locations to constrain suitable wind farm sites, and subsequently evaluated a grid of remaining potential wind farm sites using expert opinions about a host of siting factors. Li et al. [172] took a similar GIS-based approach but also assessed future wind resources under climate change. These efforts represent possible new directions for assessing WiFSS, but the lack of a standard approach makes siting factor selection and representation highly variable. For instance, Gamboa and Munda [193] accounted for candidate wind farm sites' visual pollution with secondary simulations of viewshed (in square kilometers), in contrast to the more common method of assessing visual impact using the primary opinions of experts [100,101,199]. Another example is Pambudi and Nananukul's [207] decision to collect wind speed data using questionnaire responses. These studies [72,172,173,193,206,207] show that WiFSS studies are not confined to representing siting factors with only primary or only secondary data, but also that a common standard for their combination would benefit comparisons between study approaches.

### 3.5 Data Source and Accessibility

Beyond specifying data types (i.e., primary or secondary), it is also important for WiFSS studies to specify data sources and to ensure their accessibility [93,109]. Specifying data sources is important for several reasons, firstly doing so enables the replication of similar modeling studies and findings in the same or different spatial contexts [82,126]. Replication allows scientists and other modelers seeking to execute similar research to better understand how and where to source candidate data sources for their respective studies, thus facilitating practices of knowledge transfer and data sharing for the public [110]. Secondly, action and execution based on the results of WiFSS modeling studies require their acceptance by scientists and decision makers. This acceptance is more likely if data sources and details regarding their accessibility are specified, thereby creating transparency in the research process and allowing other modelers and the public to trust a study's findings more readily [193]. Lastly, it is important to give credit to the producers or hosts of all enlisted data sources. Citation is the primary means of demonstrating how credit should be given to existing studies and their data sources, while also helping other scientists and modelers locate data sources for their own research [101,109].

This review found that although many studies indeed specified data sources for their enlisted siting factors [77,80,130,138,139], there were also studies that did not [140,157,165,196,202,203]. Table 4a and 4b shows the number of onshore and offshore WiFSS studies, respectively, that did and did not specify data sources for common siting factors for the countries with the most studies in this review (see Fig. 2). The tables suggest that not specifying data sources was more common for onshore WiFSS studies, particularly those from Greece, Iran, and Turkey, with datasets for Distance to Airports/Protected or Wildlife Areas/Roads/Urban Centers being the most frequently unspecified. There exist a few reasons why WiFSS studies may not specify their data sources, first among which is that the datasets used in these studies may be proprietary, as was often the case for studies that incorporated military zones [126,215], bird migration habitats [152,153] and protected areas [164,193] into their siting factor choices. This non-disclosure of data sources can also be for legal and/or regulatory reasons [139]. Secondly, discussions about the importance of sharing datasets for environmental model development started relatively recently; the practice of dataset sharing is crucial for asserting any modeling practice as its own discipline [110]. The importance of providing citations for secondary datasets is particularly salient for siting factors enlisted as constraints (Theme 3). Specifying these datasets means that studies performed in the same geographical context could brand the same locations as being (un)suitable for wind energy development, allowing for focused refinement of the siting factors used for evaluation and of the models themselves [124]. Specifying dataset sources for siting factors is also important for studies that employ primary datasets. Some studies in this review did not clarify how they obtained expert opinions about siting factors, nor did they provide the questions that were posed in the questionnaires or interviews conducted with them [172,188,207]. Although these data would be useful, their non-disclosure could be for ethical reasons such as protection governed by institutional review boards [182,184].

**Table 4a:** Countries with four or more studies (see Fig. 2) that specified data sources for the 15 most common siting factors in this systematic review (see Fig. 3) for onshore WiFSS studies. Each cell contains the number of studies that did and did not specify their data sources, the latter in parentheses. See the “Data Source” columns of the Supplementary Material.

	Countries (Number of Onshore Studies)						
Siting Factor	China (10)	Turkey (13)	Iran (12)	Greece (6)	United States (6)	Spain (5)	Saudi Arabia (4)
Distance to Agricultural Areas	1 (0)	1 (0)	2 (0)	3 (1)	2 (0)	1 (1)	–
Distance to Airports	1 (0)	2 (5)	2 (2)	2 (4)	1 (2)	2 (2)	1 (2)
Distance to Animal Habitats or Migration Zones	3 (1)	3 (4)	–	3 (2)	2 (0)	1 (2)	1 (1)
Distance to Historic Places	0 (1)	1 (2)	1 (2)	3 (3)	–	2 (1)	1 (0)
Distance to Protected or Wildlife Areas	3 (1)	6 (5)	5 (4)	4 (2)	4 (1)	2 (3)	1 (2)
Distance to Railroads	1 (0)	1 (2)	0 (2)	–	2 (0)	2 (1)	–
Distance to Roads	3 (2)	4 (4)	4 (3)	3 (3)	2 (3)	2 (1)	3 (1)
Distance to Transmission Lines or Substations	4 (1)	5 (4)	3 (4)	2 (/2)	2 (1)	2 (1)	2 (0)
Distance to Urban Centers	4 (2)	5 (3)	6 (4)	3 (3)	4 (2)	2 (2)	1 (2)
Distance to Water Bodies	2 (1)	4 (3)	4 (2)	1 (2)	3 (1)	2 (1)	0 (2)
Elevation	3 (1)	5 (1)	5 (2)	–	1 (1)	1 (0)	–
Life Cycle Cost	4 (0)	4 (1)	2 (4)	0 (1)	–	1 (1)	1 (0)
Slope	5 (1)	6 (3)	7 (4)	4 (1)	2 (1)	3 (1)	0 (2)
Wind Power Density	4 (1)	2 (5)	4 (0)	–	1 (0)	–	1 (0)
Wind Speed	6 (1)	8 (2)	7 (4)	4 (2)	5 (1)	2 (2)	4 (0)

**Table 4b:** Same as Table 4a but for the offshore WiFSS studies included in this systematic review.

	Countries (Number of Offshore Studies)						
Siting Factor	China (6)	Turkey (2)	Iran (1)	Greece (3)	United States (1)	Spain (0)	Saudi Arabia (0)
Distance to Airports	1 (1)	1 (0)	–	1 (2)	–	–	–
Distance to Animal Habitats or Migration Routes	3 (1)	2 (0)	1 (0)	2 (0)	1 (0)	–	–
Distance to Commercial Fishing Areas	–	1 (0)	–	–	1 (0)	–	–
Distance to Military Zones	–	2 (0)	–	3 (0)	–	–	–
Distance to Protected or Wildlife Areas	3 (0)	1 (0)	1 (0)	3 (0)	–	–	–
Distance to Shipping Lanes	2 (1)	2 (0)	1 (0)	3 (0)	1 (0)	–	–
Distance to Shipping Ports or Coastlines	2 (1)	1 (0)	1 (0)	3 (0)	1 (0)	–	–
Distance to Transmission Lines or Substations	1 (2)	2 (0)	1 (0)	3 (0)	1 (0)	–	–
Life Cycle Cost	2 (1)	1 (0)	1 (0)	1 (0)	–	–	–
Ocean Depth	3 (1)	2 (0)	1 (0)	3 (0)	–	–	–
Other Wind Properties	–	–	–	–	–	–	–
Policy Support	0 (2)	2 (0)	1 (0)	–	–	–	–
Soil or Rock Type	1 (0)	1 (0)	–	1 (0)	–	–	–
Wind Power Density	1 (2)	–	–	–	–	–	–
Wind Speed	2 (4)	2 (0)	1 (0)	3 (0)	–	–	–

Beyond specifying dataset sources, there is also the issue of data accessibility for siting factors enlisted in WiFSS models. Although most studies in this review cited their datasets [80,131,139,143,216], others only listed the names of the institutes who provided their datasets in the Methods section [46,127,133], and some did not list their datasets at all [157,165,198]. By not providing full citations with functional links to enlisted datasets, the nature of the data that informed each siting factor becomes difficult to ascertain. There was also some inconsistency in how these studies reported their dataset sources. While some studies provided data source details in the main text [139,157], other studies provided details in an Acknowledgements section [61,211], and others only in their lists of references [77,80,180]. Without a recognizable, consistent way of identifying dataset sources, it becomes harder for readers to identify what datasets informed each siting factor, as well as the data preparation that would have been necessary to incorporate them into a given WiFSS model. This review also highlighted an issue with incomplete citation for selected datasets. Even among studies that did provide citations for their dataset sources, these citations were sometimes only the name of the government website, piece of legislation, or research institute that provided the data, without specific details regarding the dataset source [127,133]. When links to dataset sources were included, they were sometimes not accessible, even in recent WiFSS studies [134,151]. Greater emphasis should be placed on ensuring that dataset sources for siting factors are fully sourced in a manner that is consistent across WiFSS studies, in order to encourage the use of publicly available, high-quality datasets, and to standardize their citation and presentation in published work.

## 4 Discussion and conclusions

Ongoing expansion of wind energy development is placing an increasing onus on siting commercial wind farms in productive, affordable, and technically feasible locations. A practice of developing and running models to assess wind farm site suitability has simultaneously grown worldwide, and consequently the need to coordinate certain aspects of independent WiFSS model development has also grown. This review paper specifically addressed how previous WiFSS studies have selected and represented their models’ siting factors, with the objectives of summarizing the similarities and differences in these siting factor choices and recommending that common standards in their selection and representation be adopted in future work. Previous review papers on similar topics have discussed siting factors in the context of their importance to WiFSS [93], the techniques used to compute, normalize, and weight selected siting factors [109], and the range of values and weights assigned to siting factors in different WiFSS studies [70]. The current review instead focuses on contrasting the subjective siting factor choices made by WiFSS model developers and how adopting consistent vocabulary, presentation styles, and implementation for siting factors could benefit WiFSS modeling as a discipline.

By performing a systematic literature review on 116 identified articles, it was found that siting factor choices in WiFSS models and the reasons for adopting common standards for them, were best discussed under the headings of five themes, summarized below. Within the summary of each theme are recommendations for how siting factor selection and representation could be standardized in future work:

1. *Deciding upon Siting Factors.* WiFSS studies frequently justify siting factors to be included in their models based on those used in prior work. Their usage in previous studies, and reasoning by model developers, has resulted in some siting factors, such as Wind Speed, Distance to Roads, and Elevation, being used more often than others. The reasoning employed by modelers to select certain siting factors over others may be motivated by the overarching study context (e.g., assessing social acceptance of wind energy development), the geographical context, the perceived importance of siting factors, or whether the study is one of onshore or offshore WiFSS. The decision to include or exclude siting factors in a WiFSS model is sometimes obvious, such as only certain study areas being vulnerable to earthquakes [201], but not explaining siting factor selections can be detrimental when comparing different studies with similar geographical contexts. For instance, Deveci et al. [229] justified including water depth in their study of offshore WiFSS in New Jersey to represent the required foundation structures and costs, but Mekonnen and Gorsevski's [183] participatory GIS model of offshore WiFSS in Lake Erie excluded water depth with no justification. Given the United States Federal government's current interest in rapidly expanding offshore wind energy in the coming years [40,41], and the limitations sea depth places on constructing new wind farms [230], model developers could increase their impact on political decision-making by using consistent sets of siting factors that raise fewer questions among non-modelers. If a decision is made to exclude siting factors frequently used in prior work, model developers have a responsibility to justify that decision explicitly.
2. *Classifying Data and Siting Factor Terminology.* While some WiFSS studies group their siting factors under the subheadings of broader classification terms (e.g., economic, environmental, social, technical, etc.), others do not, or they may use different terms that describe the same sorts of classification. The advantage of classifying is clarification of the role that siting factors play in a given WiFSS model. For example, Distance to Roads and Distance to Urban Centers represent “technical” aspects of wind energy development. Classification is especially useful when comparing WiFSS studies that utilized the same siting factors. The terminology used to describe the same siting factors (e.g., “Wind Speed” versus “Wind Potential”) is a second example of the importance of uniform vocabulary when conducting WiFSS studies. In their review of modeling water flows, Refsgaard and Henriksen [231] conclude that adopting standard terminology is important for “bridg [ing] the gap between scientific philosophy and pragmatic modelling”. The importance of language choices that have an agreed-upon definition has also been recommended by dietetic [232], ecological [233], and behavioral [234] model developers. Proposing common definitions and vocabulary for the siting factors used in different WiFSS models, and enlisting classification terms that group factors with related effects on WiFSS, should thus be a priority as this modeling discipline expands. Based on the findings of the current review, common classifications such as economic, environmental, social, and technical should be made commonplace, though with refined definitions, and uncommon names for siting factors (e.g., “Wind Blow” rather than “Wind Speed” [202]) should be avoided in future work.
3. *Implementing Siting Factors as Constraints or as Evaluation Criteria.* GIS-based approaches to WiFSS tend to incorporate siting factors as Boolean restrictions on potential wind farm locations (constraints) and/or as quantified suitability with distance or magnitude (evaluation criteria). While some siting factors are used frequently as constraints (e.g., Wind Speed, Slope, and Ocean Depth), some studies implement the same siting factors as evaluation criteria instead, or in addition to being constraints. There is also the concern that logic for the selection and setting of constraints is left unspecified in some studies, meaning that readers must guess whether, for instance, a minimum Distance to Protected Areas of 500 m is appropriate for other study contexts. Not addressing the subjectivity in setting constraints and evaluation criteria can cause serious wind farm planning risks. For example, the five WiFSS studies from Spain in the current review all enlisted a Distance to Protected Areas constraint; one study did not specify its constraint [193], three studies prohibited development within protected areas [83,122,169], and the fifth study also applied a 1000-m buffer distance to these areas [131]. Rodríguez-Rodríguez et al. [235] found that Spain's protected areas are more vulnerable to any form of land development when not surrounded by a buffer distance. For that reason, using a WiFSS study to guide wind energy development in Spain that does not adequately protect vulnerable flora and fauna could cause inadvertent environmental damage, such as heightened avian mortalities [61,62]. It is thus recommended that future WiFSS studies apply siting factors for constraint or evaluation in a manner consistent with existing literature, government policy, and expert opinions.
4. *Utilizing Primary and Secondary Data.* There are some commonly used datasets for WiFSS studies that represent siting factors with secondary data, such as the Global Wind Atlas for Wind Speed and the United States Geological Survey for Slope and Elevation. However, the familiarity of model developers with certain, often more localized, datasets can result in quite different depictions of WiFSS due to using different datasets, even within the same geographical contexts. Inconsistencies also exist in the representation of siting factors with primary data, such as the decision to involve experts in the siting factor selection process, whether to use a linguistic scale to capture experts' opinions, and the frequent differences in enlisted siting factors. This review found that the decision to use primary and/or secondary data in WiFSS studies often depended on the research question being answered. Studies that focused on developing a model that could construct a continuous WiFSS surface were more likely to rely solely on secondary data, such Mann et al.‘s [212] logistic regression-based approach to assessing WiFSS in Iowa. By contrast, primary data were most often incorporated in WiFSS studies that ranked candidate wind farm sites for their development potential based on expert opinions, as in Deveci et al.‘s [236] assessment of potential offshore wind farm locations in Norway. The innovative methods of integrating primary and secondary data presented by this review were of particular interest. Standout examples include Rezaei-Shouroki et al.‘s [206] use of expert opinions to construct a land cost siting factor alongside secondary sources for population and wind speed, and Xu et al. [72] using secondary spatial data layers to constrain potential sites and then using expert opinions to rank the remaining sites. WiFSS approaches that combine primary and secondary data sources should continue to be pursued, using these existing studies as a basis for standardizing how their siting factors are represented.
5. *Data Source and Accessibility.* Facilitating common standards for siting factor selection in WiFSS studies depends highly on providing complete and functional citations for enlisted datasets. This review showed, however, that some WiFSS studies do not provide any citation for enlisted secondary datasets; some only specify the dataset provider in-text without a full citation, and sometimes citations lack specific details on how to access the data sources used in these studies. Some WiFSS studies that represented siting factors with primary data also did not fully detail their data sources, namely how expert opinions about candidate wind farm sites were collected or the questions that were asked of said experts. At a time when scientific integrity is being questioned by the public and elected officials [237], there is an increasing onus upon model developers to ensure that their work is fully transparent and accessible, such as by making effective use of free online repositories [238] and preparing robust model documentation [239]. This motion toward transparency in part requires documenting how siting factors were selected, and how and where each factor's dataset(s) can be found in a way that both modelers and non-modelers can recognize across separate studies. While proprietary knowledge and institutional review boards often limit the extent to which data for siting factors can be shared, a standard presentation of dataset details in-text, and providing complete citations, would allow modelers to share knowledge of robust datasets more easily, while also garnering trust in their work.

The intent of this review paper is not to dictate how siting factors in WiFSS models should be selected and represented moving forward. Rather, this review paper serves to identify that an explicit common standard for siting factor selection and representation does not yet exist, and that a set of standards for siting factors could be adopted by at least recognizing the role played by subjective modeling decisions (e.g., approach to citation, deciding upon important siting factors, siting factor vocabulary). As the wind energy sector continues to grow, so will the demand for WiFSS modeling research, and consequently the need to describe the same determining factors for wind energy development to new, larger audiences. Future WiFSS studies will ideally use the trends in siting factor application presented by the thematic synthesis, and the recommendations derived from each theme, to inform approaches toward siting factor selection and representation that converge throughout subsequent studies toward a common approach.

## Author contribution statement

All authors listed have significantly contributed to the development and the writing of this article.

## Data availability statement

Data included in article/supplementary material/referenced in article.

## Declaration of competing interest

The authors declare that they have no known competing financial interests or personal relationships that could have appeared to influence the work reported in this paper.
